# Fluorescence-suppressed time-resolved Raman spectroscopy of pharmaceuticals using complementary metal-oxide semiconductor (CMOS) single-photon avalanche diode (SPAD) detector

**DOI:** 10.1007/s00216-015-9156-6

**Published:** 2015-11-09

**Authors:** Tatu Rojalin, Lauri Kurki, Timo Laaksonen, Tapani Viitala, Juha Kostamovaara, Keith C. Gordon, Leonardo Galvis, Sebastian Wachsmann-Hogiu, Clare J. Strachan, Marjo Yliperttula

**Affiliations:** Division of Pharmaceutical Biosciences, Centre for Drug Research, University of Helsinki, P.O. Box 56, 00014 Helsinki, Finland; Department of Pathology and Laboratory Medicine, and Center for Biophotonics, University of California Davis, 2700 Stockton Blvd, Sacramento, CA 95817 USA; Faculty of Information Technology and Electrical Engineering, Department of Electrical Engineering, University of Oulu, P.O. Box 4500, 90014 Oulu, Finland; Department of Chemistry, MacDiarmid Institute for Advanced Materials and Nanotechnology, University of Otago, Union Place West, Dunedin, 9054 New Zealand; Division of Pharmaceutical Chemistry and Technology, University of Helsinki, Viikinkaari 9, 00014 Helsinki, Finland; School of Chemical Technology, Department of Forest Products Technology, Aalto University, P.O. Box 16300, 00076 Helsinki, Finland

**Keywords:** Raman, Time resolved, CMOS SPAD, Pharmaceuticals, Fluorescence suppression, Process analytical technology (PAT)

## Abstract

In this work, we utilize a short-wavelength, 532-nm picosecond pulsed laser coupled with a time-gated complementary metal-oxide semiconductor (CMOS) single-photon avalanche diode (SPAD) detector to acquire Raman spectra of several drugs of interest. With this approach, we are able to reveal previously unseen Raman features and suppress the fluorescence background of these drugs. Compared to traditional Raman setups, the present time-resolved technique has two major improvements. First, it is possible to overcome the strong fluorescence background that usually interferes with the much weaker Raman spectra. Second, using the high photon energy excitation light source, we are able to generate a stronger Raman signal compared to traditional instruments. In addition, observations in the time domain can be performed, thus enabling new capabilities in the field of Raman and fluorescence spectroscopy. With this system, we demonstrate for the first time the possibility of recording fluorescence-suppressed Raman spectra of solid, amorphous and crystalline, and non-photoluminescent and photoluminescent drugs such as caffeine, ranitidine hydrochloride, and indomethacin (amorphous and crystalline forms). The raw data acquired by utilizing only the picosecond pulsed laser and a CMOS SPAD detector could be used for identifying the compounds directly without any data processing. Moreover, to validate the accuracy of this time-resolved technique, we present density functional theory (DFT) calculations for a widely used gastric acid inhibitor, ranitidine hydrochloride. The obtained time-resolved Raman peaks were identified based on the calculations and existing literature. Raman spectra using non-time-resolved setups with continuous-wave 785- and 532-nm excitation lasers were used as reference data. Overall, this demonstration of time-resolved Raman and fluorescence measurements with a CMOS SPAD detector shows promise in diverse areas, including fundamental chemical research, the pharmaceutical setting, process analytical technology (PAT), and the life sciences.

**Electronic supplementary material:**

The online version of this article (doi:10.1007/s00216-015-9156-6) contains supplementary material, which is available to authorized users.

**Graphical abstract:**
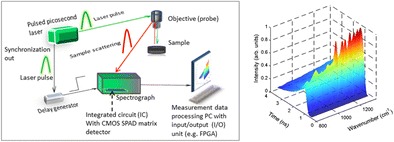
Time-resolved Raman measurement of a pharmaceutical sample using the complementary metal-oxide semiconductor (CMOS) single photon avalanche diode (SPAD) detector technology

## Introduction

Raman spectroscopy is beneficial for diverse types of quantitative and qualitative analyses in fundamental chemical research [1], life sciences [2, 3], and pharmaceutical research and development, for example during dosage form manufacturing, storage, and administration [4, 5]. Other disciplines include material analysis and mineralogy. However, major limitations to its use have included photoluminescence frequently masking the Raman signal [6], weak Raman scattering, and insufficiently sensitive detectors. Some of these problems have been resolved with improvements in hardware, such as the development of efficient, low-noise, silicon-based array detectors, stable high-power diode lasers, and high-resolution spectrometers [7]. Thus, Raman has become widely applied in both basic and applied chemistry. Despite the significant technical advances, the weak signal and simultaneously strongly interfering background from photoluminescence are still hindrances in Raman spectroscopy. This is particularly problematic in the biomedical, life science, and pharmaceutical settings where the analysis of biological samples and drug molecules typically suffers from strong photoluminescence backgrounds. At the same time, there is an unquestionable need for label-free technologies with sufficient temporal resolution for the detection of in situ real-time phenomena. Such measurements include, for example, phase transformation analysis of various drugs and experiments with living cells [8].

The photoluminescence (PL) phenomena can be divided into various types according to the specific molecular relaxation sequences that take place after the absorption of an incident photon [9]. However, fluorescence is usually the main component of photoluminescence and also the one that overlaps spectrally with the Raman spectrum, making it very difficult to remove from Raman measurements. Strong fluorescence is typically created in conjugated double bonds and polycyclic aromatic rings, which are common structures in many drugs and biological molecules. This fluorescence frequently occurs on the same wavelength range as the Raman fingerprint in a given experiment, and as fluorescence is usually a much stronger phenomenon, it can completely overwhelm the Raman signal. The means to alleviate this fluorescence problem in Raman spectrometry can be essentially divided into continuous-wave (CW) and time-resolved approaches. In addition, various algorithm-based methods relying on the spectral post-processing have been introduced. In order to clarify why these methods are typically used, several statements related to photochemical and physical mechanisms of photoluminescence phenomena are needed. Herein, we assume that a laser with the center wavelength *λ*_L_ is used as the excitation source. Firstly, the Raman phenomenon is an inelastic scattering process present at all excitation wavelengths, whereas fluorescence requires a nonzero electronic molecular absorption coefficient at *λ*_L_. The intensity of Raman scattering is approximately proportional to $\frac{1}{\lambda_{\mathrm{L}}^4}$. Thus, as a rule of thumb, shorter wavelength lasers give rise to stronger Raman signal intensities. Secondly, the spectral shape of fluorescence from a collection of identical fluorophores is independent of *λ*_L_ while the Raman spectrum as a whole is moved to shorter or longer wavelengths along with the excitation. Thirdly, the temporary storage of energy within the molecule after the absorption and relaxation processes entails a characteristic statistical average delay (having an exponential distribution with a certain expectation value) before the emission of a fluorescence photon. This is manifested as the fluorescence “tail” after a short excitation pulse. Theoretically, Raman scattering is an instantaneous process, and it does not involve such delays. However, significant temporal broadening can be observed, which occurs in thick scattering samples; it could be so that the analyte molecules deeper in the sample will interact with the excitation photons even hundreds of picoseconds later than the molecules on the surface [10, 11]. Methods to determine the decay rate of Raman scattering have also been introduced [12]. It turns out that the time dimension provides, in a sense, the most general way to deal with fluorescence in Raman spectrometry. In the following, the CW and time-resolved approaches are briefly described.

In 1976, Funfschilling and Williams resolved the fluorescence-related problems for the first time [13]. They exploited the previously known method of phase sensitive detection that had been effectively applied in nuclear magnetic resonance (NMR), electron paramagnetic resonance (EPR), and photoelectron spectroscopy. The common basis for those methods was to shift the signals of interest away from the interfering background. Thus, by modulating the Raman excitation wavelength, the Raman signal could be recovered from the overlapping fluorescence signal. Later on, a wavelength differential technique was developed and became known as shifted excitation Raman difference spectroscopy (SERDS) [14]. To some extent, differential techniques are applicable to separate fluorescence and Raman signals [15]. Noteworthy approaches include frequency and polarization modulations. The advantage of these differential methods lies in the possibility to separate the non-shifted continuous-wavelength (CW) fluorescence and Raman signals in a quantitative way. However, in general, these techniques have their limitations, for example acquiring two separate spectra for post-processing in order to resolve the Raman signals.

Since the Raman intensity is proportional to the fourth power of the excitation laser’s frequency, the shorter wavelength (i.e., higher frequency) lasers give rise to a stronger Raman signal intensity. However, increasing the excitation photon energy increases also the likelihood of a sample to contain electronically excitable chromophores, each one of which may contribute to fluorescence. Conversely, decreasing photon energy decreases the number of such chromophores. Using *λ*_L_ in the NIR range (typically 785–1064 nm) often makes the fluorescence-to-Raman signal ratio decrease markedly or even vanish. FTIR-Raman may be considered the gold standard here. The downsides include the required detector technology (e.g., InGaAs) having a high dark current, which, combined with low Raman efficiency, leads to a reduced signal-to-noise ratio (SNR) [16]. The required high laser powers can also lead to sample damage and destruction by heating. Using *λ*_L_ in the UV range (<400 nm) has two advantages: Raman efficiency is high and while fluorescence might be present, it resides in the longer (VIS) wavelengths and thus does not overlap with the Raman spectrum. However, the phenomenon that UV excited Raman does not suffer from fluorescence since it occurs in a different wavelength range is only true for excitation wavelengths in the deep UV below 260 nm. The downsides include the generally increased difficulty in making optics, lasers, detectors, and high-resolution spectrographs for UV, along with the sample degradation due to high photon energy compared to the VIS range [7]. Using algorithms solely without any instrument-based means to subtract the fluorescence background also has drawbacks. For instance, even though the fluorescence profile of the measured sample is relatively recognizable and smooth, a subtraction algorithm produces only an estimate on fluorescence; relevant spectra features may be subtracted, thus biasing the result. Moreover, an additional problem with subtracting fluorescence is that the shot noise will remain. Finally, using photobleaching as a means to exterminate the fluorescence-generating chromophore groups of the molecules can be slow and cause physical changes like burning to the samples. In addition, photobleaching is not a suitable technique for living organism- and cell-based assays.

Next, we consider the time-resolved approach to Raman spectrometry. Several different types of time-gated Raman systems has been demonstrated, among others those based on an intensified charge-coupled device (ICCD) [17], a streak camera, and an optical Kerr gate [18]. The ICCD can reach <100 ps time gate width, and the full optical Kerr gate, combined with a suitable laser, can reach time resolution of a few picoseconds or better. The main drawbacks of these systems compared to the current system based on a complementary metal-oxide semiconductor (CMOS) single-photon avalanche diode (SPAD) matrix and microchip laser are related to the inherent complexity, bulkiness, and/or cost. Instead, the technology at hand in principle enables the development of robust, compact, and affordable time-gated Raman spectrometers with <100 ps time resolution. This follows from the facts that the CMOS SPAD detector does not require extreme cooling unlike scientific CCDs, and the laser technology is also potentially compact. Therefore, this technology offers great promise as practical instrumentation and replacement of CW Raman systems not only in the field of pharmaceuticals but also in general.

**Fig. 1:**
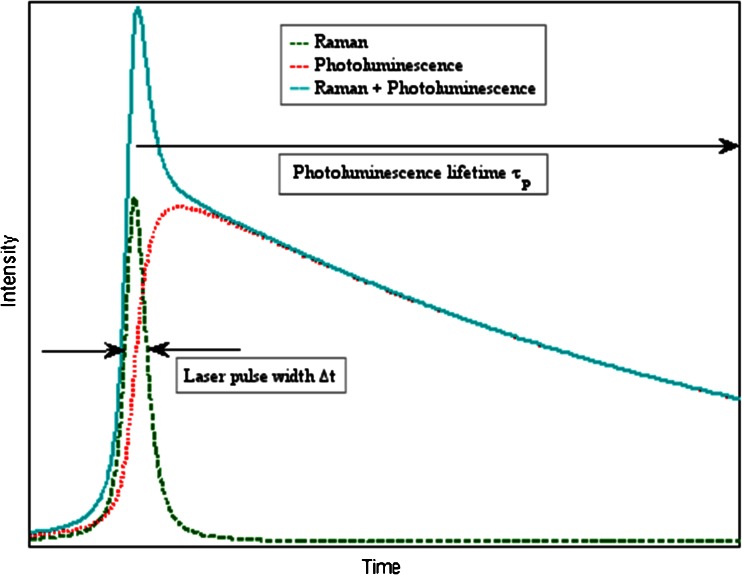
Schematic representation of the laser excitation, Raman, and photoluminescence signals

A schematic temporal shape of a Raman band with a PL background is shown in Fig. 1. The curves represent schematically the time evolution of the Raman, PL, and total emission intensity following an incident excitation pulse. The elastic part (Rayleigh scattering and Fresnel reflection) has not been depicted. As mentioned before, the Raman process differs from the luminescence mechanisms with respect to the nature and magnitude of the associated delays between photon incidence and emission. The typical time scales for Raman are in the sub-picosecond to picosecond range [12, 19], whereas those of fluorescence (the fastest spontaneous PL process) are in the 10-ps to 100-ns range [9]. Thus, if we take the laser pulse width *Δt* = 150 ps, the shape of the depicted Raman pulse is very close to that of the excitation pulse apart from a scaling factor. Using a fast photodetector such as a SPAD, one can choose to record only the part of the emission pulse containing the Raman pulse, in other words rejecting the PL tail. Essentially, the associated photon noise is also rejected—and this is the one fundamental advantage of a time-resolved Raman measurement over any possible CW Raman measurement, including differential techniques [20]. However, careful inspection of Fig. 1 and the actual measurement data shows that with our 150-ps excitation pulse, some luminescence is emitted already during the pulse. This part of the PL we henceforth call residual fluorescence (RFL). These issues will be discussed in more detail later in this article.

In the present study, we demonstrate for the first time the use of a novel detector system based on CMOS SPAD technology for the study of several pharmaceutical compounds. Here, we utilize the power of combining a picosecond pulsed laser with the time-resolved detection system to efficiently reject the fluorescence and record detailed Raman spectra for those compounds. Using a fast CMOS SPAD detector, we are able to choose and to record only the part of the emission pulse containing the Raman scattering by rejecting the fluorescence tail. The drug molecule response is recorded during the short laser pulses, and the CMOS SPAD detector is synchronized with the laser pulse. However, typically, a part of fluorescence, RFL, temporally overlaps the Raman signal and cannot be rejected by time gating. To overcome the RFL, we present a convenient algorithm to enhance the accuracy of acquired Raman spectra. We use a time-gated Raman to measure the Raman spectra of four pharmaceutical compounds. These were selected to include caffeine, as an easy reference compound; ranitidine HCl as a previously less studied and difficult compound; and indomethacin in both amorphous and crystalline forms, as particularly difficult ones suffering from strong fluorescence issues. The capabilities of the new setup were explored and compared to a conventional CW Raman instrument.

## Materials and methods

### Materials

The studied drugs were caffeine anhydrate (Orion Pharma, Helsinki, Finland), ranitidine hydrochloride polymorphic form II (Hawkins Pharmaceutical Group, Minnesota, USA), and indomethacin (crystalline γ-form, Hangzhou Dayangchem, China). The molecular structures of the studied compounds are presented in Table 1. Amorphous indomethacin was prepared by heating the crystalline powder in an aluminum dish for ∼15 min. All the other chemicals were measured as received. In conventional Raman measurements, the compounds were measured in sample holders with the powdered drug layer height of approximately 2 cm. In the time-resolved Raman measurements, a smaller amount of drug (approximately 100–200 mg) was placed onto a sample holder. The laser beam was directed onto the samples from above with both setups.

**Table 1:**
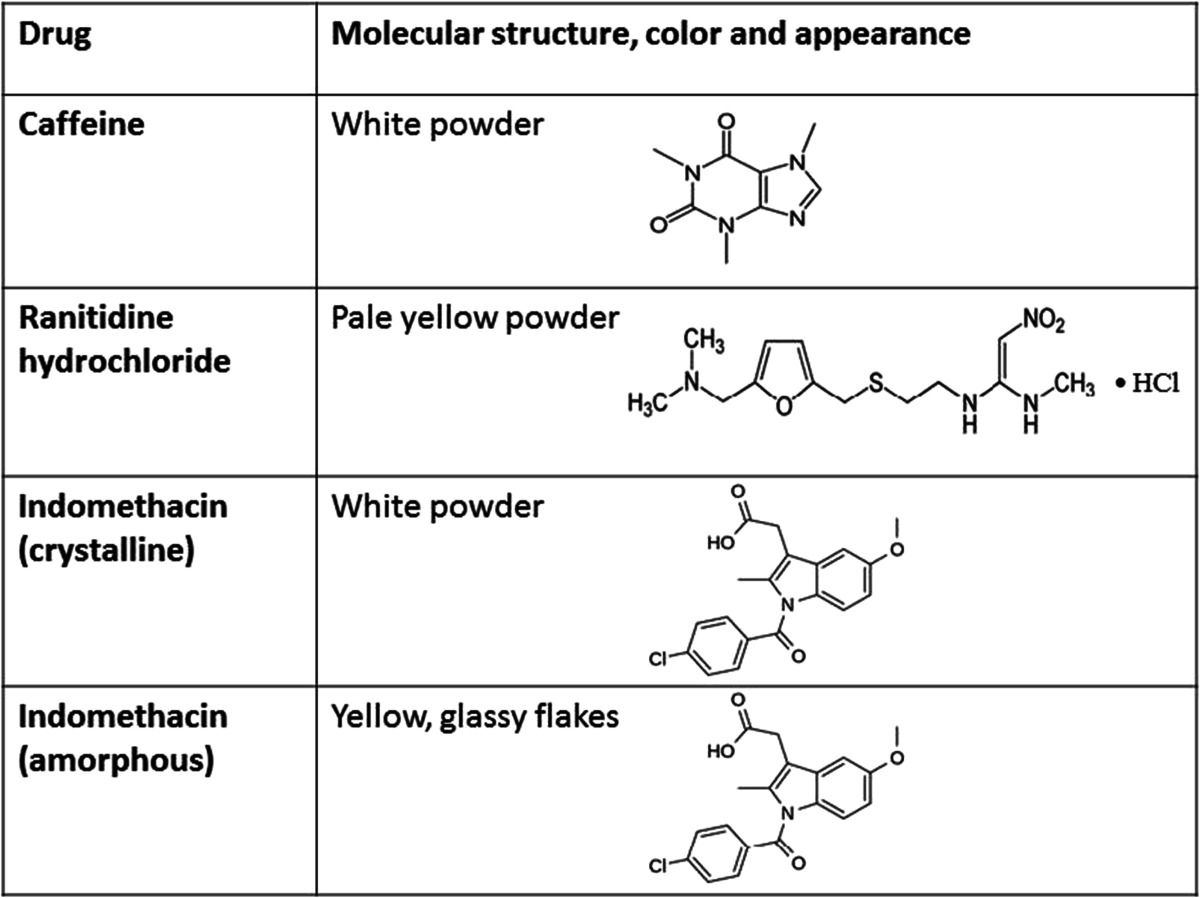
The chemical structures and physical characteristics of the studied compounds

### Raman measurements at 785 nm

The Raman measurements at 785 nm were performed using a CW Raman RXN1-PhAT-785-D-laser Invictus 785 nm (Kaiser Aerospace & Electronics Company, USA). The probe was PhAT System Probehead (Kaiser Optical Systems, Inc., USA), with collimated incident radiation and a sampling diameter of approximately 6 mm. The average laser power on the sample was ∼20 mW. The detector was a charge-coupled device (CCD), 1024 × 256 EEC MPP Type (Kaiser Optical Systems, Inc., MI, USA) cooled to −70 °C. The measurements were performed at ambient temperature and humidity, with dark background subtraction. Raman shift calibration was performed using cyclohexane. The CW 785-nm Raman spectra presented in this paper are an average of ten scans, each with an acquisition time of 3 s and a spectral resolution of approximately 4 cm^−1^.

### Raman measurements at 532 nm

A continuous excitation laser beam was focused down to a micrometer-sized spot on standard substances through a confocal Raman microscope. A frequency doubled Nd:YAG, 532 nm linear polarized excitation laser (∼20 mW) was used in combination with ×20 (Nikon, NA 0.40) and ×10 (Nikon, NA 0.25) microscope objectives. The spectra were acquired using a CCD camera (Andor Newton DU970-BV, Andor Technology plc, Belfast, UK) behind a grating (600 g mm^−1^) spectrograph with a spectral resolution of 2–3 cm^−1^. Integration times of the signal were 0.5, 1, 2, and 5 s.

### Time-gated picosecond Raman

The time-resolved measurements were performed using a laboratory setup with the following specifications: The excitation source was a custom-made 532-nm Nd:YVO microchip pulsed laser. The average power used was 14 mW, repetition rate 40 kHz, pulse width 150 ps, focus diameter 50 μm, pulse energy 0.35 μJ, peak power 2 kW, and maximum irradiance 28 MW cm^−2^. To alleviate photobleaching effects, the focal point was periodically moved over an area of about 1 mm^2^ by utilizing a custom-made galvo-mirror arrangement. The spectrograph slit was 50 μm, with ×1.7 magnification, 3.4 nm mm^−1^ dispersion, and about 13 cm^−1^ spectral resolution. The detector was the 128 × 8 CMOS SPAD matrix detector developed by Kostamovaara’s group in the University of Oulu. The detector accumulates single-photon events in an internal time histogram consisting of four bins. Only one of these bins was used as the “time gate” in the data analysis presented below. The instrument response function (IRF) is dominated by the laser pulse shape, SPAD response shape, and time gate width of the electronic width generator. The FWHM of the IRF, which is a measure of the system’s time resolution, was approximately 250 ps. This approximation has been demonstrated earlier in the literature [16]. The time-resolved spectral datasets were collected by sequentially moving the gate in 50-ps steps using the electronic delay generator. The data acquisition and setup control was maintained by a digital microchip, field-programmable gate array (FPGA). Data acquisition software and setup control were carried out by in-house programs, which were coded using LabVIEW (National Instruments Corporation, TX, USA) language. The data was processed and 3D graphics were plotted using Matlab (MathWorks, MA, USA). The measurements were performed at ambient temperature and humidity, and spectra were the sum of 1.2 × 10^8^–1.7 × 10^9^ pulses corresponding to overall measurement times in the range of 1–12 h. The signal level (counts/laser pulses/pixel) was kept at the suitably low level of a few percent at maximum to avoid signal distortion by photon counting pile-up effects. It is to be noted that the acquisition of a time-gated Raman spectrum alone, without the need to collect the whole temporal shape using a 50-ps step size, can be done in a fraction of the time mentioned above, for instance in the order of minutes or seconds. In future versions of the hardware, larger detectors and adjustable laser repetition rates enable bringing the measurement times to a level that is practical in even field and industrial process applications. The schematic illustration of the Raman and fluorescence events is presented in Fig. 1.

### Residual fluorescence background subtraction

The RFL subtraction was performed by assuming that the fluorescence is the only contributing factor in the “valleys” of the time-resolved Raman spectra. That signal can then be used to estimate the contribution of fluorescence to the signal intensity in the same time range as the Raman scattering (i.e., where the Raman peaks occur). Unfortunately, the acquired Raman data can be difficult to use for this, as the valleys are at varying heights due to baseline shift and no simple control value can be chosen. In order to get data to be internally comparable, the whole Raman spectrum was first baseline corrected at each time step using a built-in function (msbackadj) of Matlab. Next, a control value was chosen as the control valley area where it was assumed that the signal is pure fluorescence with no Raman scattering. For better representation of fluorescence, an average signal from three adjacent wavenumber bins was used. The pure Raman spectra were obtained by eliminating the amount of fluorescence by subtracting the control valley from the overall time-resolved response signal.

### Fluorescence decay estimations

The fluorescence decay estimations were performed assuming that the tested compounds do not have multiple fluorescent components. Thus, a single-exponential decay model, which fits well for gross estimations performed herein, was chosen for the fluorescence decay estimations. Furthermore, the fluorescence decay estimations were based on the assumption that there is only negligible delay between photon absorption and the molecule’s relaxation to the lowest vibrational level in the first excited electronic state before transitioning to the electronic ground state by emitting a photon (fluorescence) and non-radiatively (internal conversion) with the rate constants *k*_F_ and *k*_nr_, respectively. We used a single-exponential decay model based on Eqs. (1) and (2):

1 $$ N(t)={N}_0{e}^{-\left({k}_{\mathrm{F}}+{k}_{\mathrm{nr}}\right)t}, $$

in which *N*_0_ is the population of excited molecules immediately after a very short (delta function) laser pulse.

2 $$ {\varPhi}_{\mathrm{F}}(t)={k}_{\mathrm{F}}N(t), $$

in which *Φ*_F_ is the instantaneous fluorescence intensity (photons per unit time) at time *t*. The overall response function of the instrument is essentially a convolution of the shape of the laser pulse, the IRF, and the exponential decay shape that is described by the characteristic photoluminescence behavior of the sample. To perform the fluorescence decay time estimations for the samples, single-exponential fitting was done to the tail part of the collected data using Eqs. (1) and (2), where applicable. Ideally, the influence of the laser pulse and IRF would be differentiated from the exponential part. However, instead of attempting a de-convolution process to separate the exponential part from the effect of the laser pulse and IRF, estimates of the prospective error were performed with approximate simulations that are described in the “Discussion” section.

### Quantum chemical calculations

Density functional theory calculations were performed for vibrational mode assignment of the ranitidine HCl spectra (modeled as ranitidineH^+^). Molecular optimization and vibrational frequency calculations were performed with Gaussian 09 [21] software using density functional theory (DFT), and the B3-LYP functional with the 6-31G(d) basis set. The predicted vibrational modes were scaled with the recommended factor of 0.9614 [22] using GaussSum 2.2 [23] software and visualized using GaussView (v. 5.0, Gaussian, Inc. Wallingford, CT, USA).

## Results

### CW Raman measurements at 785 and 532 nm

**Fig. 2:**
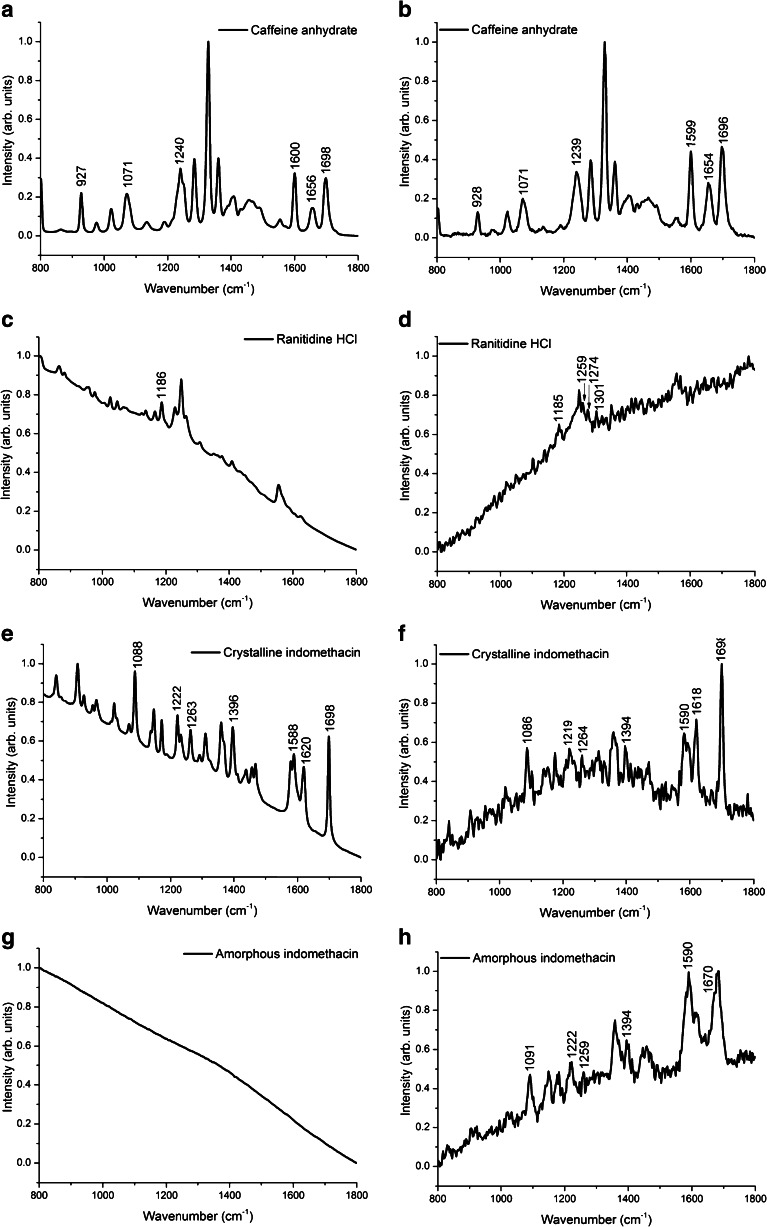
The Raman spectra obtained using the CW 785-nm Raman spectrometer for caffeine (**A**), the CW 532-nm Raman spectrometer for caffeine (**B**), the CW 785-nm Raman spectrometer for ranitidine hydrochloride (**C**), the CW 532-nm Raman spectrometer for ranitidine hydrochloride (**D**), the CW 785-nm Raman spectrometer for crystalline indomethacin (**E**), the CW 532-nm Raman spectrometer for crystalline indomethacin (**F**), the CW 785-nm Raman spectrometer for amorphous indomethacin (**G**), and the CW 532-nm Raman spectrometer for amorphous indomethacin (**H**). All spectra are normalized from 0 to 1

The conventional CW Raman spectra of the model drugs were measured in the region 800–1800 cm^−1^. This spectral range encompasses the informative Raman features in both the fingerprint and group frequency regions. The CW Raman spectra are shown in Fig. 2A–H. In the Electronic Supplementary Material (ESM) Tables S1–S4, the Raman peaks for every drug are assigned between 800 and 1800 cm^−1^, based on the quantum chemical calculations published previously or performed in this study. In the tables, selected peaks are assigned for CW non-time-resolved measurements and time-resolved measurements. The spectra have been normalized from 0 to 1, and in order to represent raw spectra, they have not been smoothed.

Panels A and B of Fig. 2 show the Raman spectra of caffeine for CW 785-nm excitation source and CW 532-nm excitation source, respectively. Very low fluorescence intensity can be observed, and the Raman peaks can be easily recognized. Panels C and D of Fig. 2 show the acquired CW 785- and 532-nm Raman spectra for ranitidine hydrochloride. A relatively large fluorescence background hampers the interpretation of both, and only a handful of weak Raman peaks really stand out. In Fig. 2E, F, for the crystalline form of indomethacin, a fluorescence background is present, but observable Raman peaks can be separated with both setups. Using the CW 785-nm excitation source for amorphous indomethacin, Fig. 2G, does not show any spectral details as fluorescence dominates the whole spectrum. As observed in Fig. 2H, the experiment with the CW 532-nm setup reveals more interpretable spectral features.

On the basis of these CW measurements, all the samples had undefined background increase, indicating photoluminescence, except for caffeine. Using the CW 785-nm setup, the most fluorescent sample appears to be amorphous indomethacin, followed by ranitidine hydrochloride and gamma crystalline indomethacin. However, with the CW 532-nm instrument, the most fluorescent drug is ranitidine hydrochloride, followed by amorphous indomethacin and then gamma crystalline indomethacin.

### Time-resolved Raman measurements

**Fig. 3:**
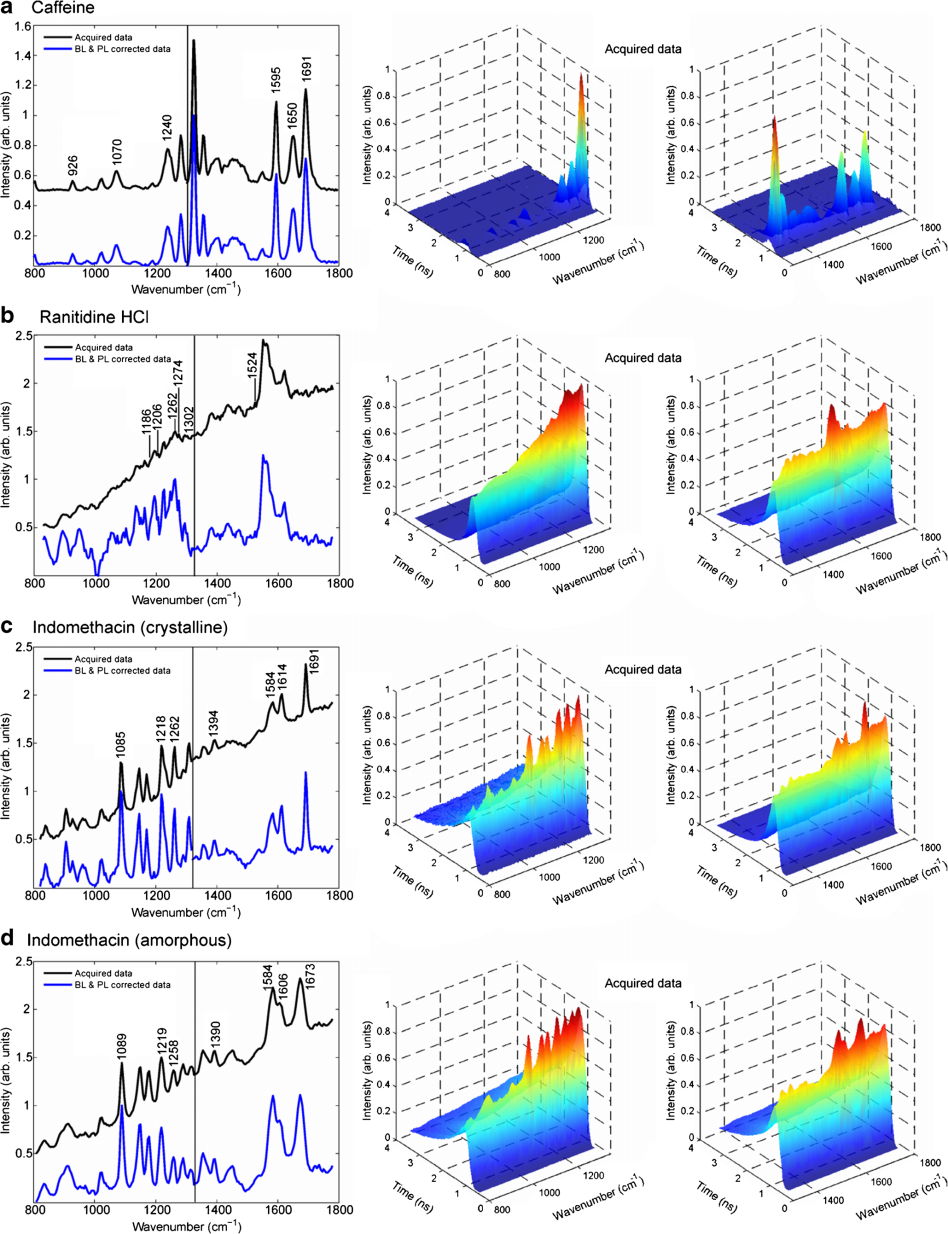
Time-resolved Raman spectra. *Panels* represent the 2D, traditional Raman spectra (*left*) and the 3D spectra (*right*). The 2D plots are reconstructed from two separate measurements after normalization of the data. The *vertical line* separates the two measurements. The acquired, unprocessed raw data is shown in the 3D plots to give a clear view of the events in the time domain after the laser pulse; the initial rise of signal after the laser pulse consists mainly of Raman scattering, and the settling of the signal consists of the decaying fluorescence signal. The drugs shown in the *panels* are **A** caffeine, **B** ranitidine hydrochloride, **C** indomethacin (crystalline), and **D** indomethacin (amorphous)

The time-resolved spectra for the model drugs are represented in Fig. 3A–D. In the panels, black lines correspond to acquired raw data, blue spectra represent the baseline- and photoluminescence-corrected data, and the 3D representations show the evolution of the measured signal in time dimension. The measurement region was 800–1800 cm^−1^. The time-resolved spectra (Fig. 3A–D) were recorded separately for the regions 800–1300 and 1300–1800 cm^−1^. The reason for this is related to the length difference between the detector array and the spectrum produced by the spectrograph in the current setup. The spectra have not been smoothed.

**Fig. 4:**
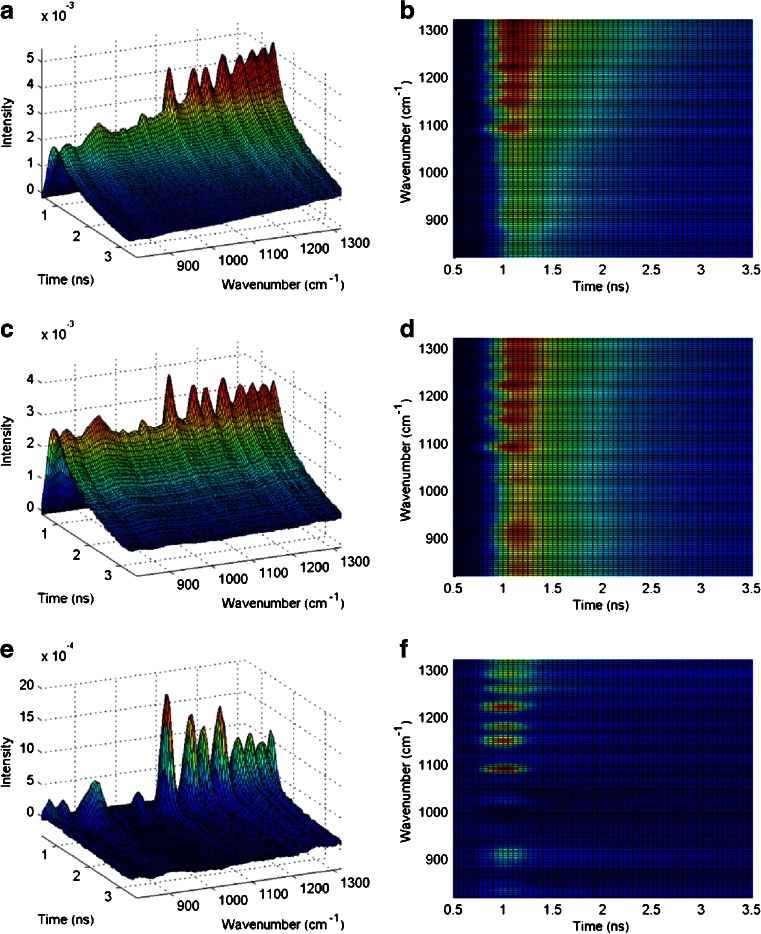
Acquired Raman data from amorphous indomethacin (**A**, **B**), baseline-corrected data (**C**, **D**), and fluorescence-subtracted data (**E**, **F**). *Panels* on the *left* illustrate the intensity vs. time measurements for each wavenumber shown as a 3D stack. *Panels* on the *right* are also the *top views*

Figure 3A illustrates the time-resolved Raman spectrum for caffeine. Very little fluorescence is observed. However, for ranitidine HCl and both forms of indomethacin (Fig. 3B–D), the fluorescence background is substantially reduced, and more Raman details in the spectra can be seen. As described earlier in this text, the residual fluorescence (RFL) can be subtracted with simple data manipulation. This is demonstrated below for amorphous indomethacin as an example. Figure 4A, B illustrates the acquired raw data. In the next step, the baseline correction is performed, as shown in Fig. 4C, D. A control valley at 1056 cm^−1^ was chosen as the control area where it is assumed that the signal is pure fluorescence with little or no Raman photons. As the shape of the fluorescence does not change much in time, the contribution of this control valley was subtracted from the overall response signal, Fig. 4E, F. The method was able to isolate the Raman peaks, as the 3D spectrum is now homogenously flattened everywhere except the Raman peak areas. In particular, the region between 950 and 1250 cm^−1^ becomes clear with this treatment and four strong and sharp Raman peaks can be easily seen. The estimated decay time for the control valley was the same as the decay time estimated for the raw data. Therefore, the spectral treatment was able to preserve the shape of the fluorescence signal and to isolate the fluorescence free time-resolved Raman response. In summary, this straightforward data processing enhances the interpretation possibilities even further, as the blue spectra show in Fig. 3A–D.

With the present CMOS SPAD detector, three different factors are simultaneously defined: the time distribution of detected photons (Fig. 3A–D: the 3D panel representations; time axis), the photon intensity (Fig. 3A–D: the 3D panel representations; intensity axis), and the energies of photons that arrive at the detector (Fig. 3A–D: the 3D panel representations; wavenumber axis). Thus, an informative representation of photon distribution is obtained as represented in the panels. The 3D representations show the exact starting point of the increase in Raman signal immediately after the laser pulse, as well as the fluorescence rise and decay. For example, in Fig. 3D for amorphous indomethacin, in the time domain, the maximum signal from Raman scattering occurs shortly after the laser shot (yellow- and red-colored peak regions). Moreover, the fluorescence appears to start dominating the response signal at ca. 2.5 ns (blue, decaying slope). It is worth stressing that in the case of relatively thick scattering samples, temporal broadening of Raman signals may have contribution to the time at which the fluorescence starts dominating the response signal [10, 11]. Importantly, this is a sample-specific phenomenon and depends on the sample’s fluorescence. The time-domain analysis can be utilized to estimate the fluorescence decay times, which we introduce in the “Discussion” section. Another observation that can be made in the spectra of Figs. 2 and 3 is that the shape of the fluorescence background is negative for the measurements recorded with CW 785-nm excitation, changing to a positive slope in the time-resolved measurements. This effect can be attributed to the different excitation wavelength and to the fact that the NIR experiment is performed at the edge of the sensitivity curve of the CCD detector.

## Discussion

### Fluorescence rejection with the pulsed picosecond laser and the CMOS SPAD detector

The time-resolved setup used in this study has a 532-nm pulsed laser, and it is combined with the CMOS SPAD detector having relatively fast electronic gating possibility to temporally resolve the Raman spectrum from the fluorescence background. An example can be seen with the amorphous form of indomethacin, which is yellow in color. With the non-time-resolved CW 785-nm setup, it was not possible to detect any Raman peaks due to the fluorescence, and another CW 532-nm instrument had to be used to acquire interpretable Raman spectrum. With the time-resolved CMOS SPAD system, the analysis could be conveniently done at once for all the model compounds. Furthermore, the other benefits of Raman spectroscopy, such as minimal sample preparation, significant amount of chemical information, and lack of sensitivity to sample water content, are preserved. Combined with the fluorescence-suppressed Raman spectra, the time-domain studies could be a useful tool in Raman spectroscopy to evaluate various changes in a system such as phase transitions or changes in the chemical content of the medium in cell cultures. At the end of this discussion, we preliminarily address the potential of exploiting the time-dimension analysis parallel to the fluorescence-suppressed Raman data. The effect of time resolution is shown in Fig. 3; the spectra are shown as 2D and 3D representations. The 2D spectra represent the spectrum with the highest Raman scattering intensity collected after the laser shot, i.e., it corresponds to one specific time point in the 3D spectrum. This is close to the CW Raman spectra in appearance (Fig. 2), and in terms of signal-to-noise ratio, it is clearly better. On the other hand, if one is interested in the Raman and fluorescence signals’ time evolution, the 3D data show the stack of spectra from moments before the emission pulse, the rise of Raman and fluorescence signals, and the decay of fluorescence. Further spectral treatments with simple algorithms can be used to fully eliminate the contribution of residual fluorescence (Fig. 3: BL- and PL-corrected data, Fig. 4) from the time-resolved spectra. It is noteworthy that the time-resolved raw spectra alone are able to show Raman details on all the tested compounds. This makes the instrument potentially able to, for example, identify compounds or different solid-state forms. However, in accurate quantitative analysis, the algorithm-treated data can be more useful.

### Analysis of the measured drugs—new possibilities with the time-resolved Raman

The Raman shifts for selected peaks from the CW 785-nm, CW 532-nm, and the time-resolved spectra are shown in ESM Tables S1–S4. For all crystalline samples, the band positions are in close agreement. Raman band assignments have been previously published using DFT calculations for caffeine and crystalline and amorphous indomethacin, and these are also included in ESM Tables S1, S3, and S4. Computational chemistry-based band assignments have not previously been published for ranitidine hydrochloride, and thus, density functional theory calculations have been included in this study (modeled as ranitidineH^+^) and prominent bands assigned in ESM Table S2, together with their corresponding eigenvector diagrams in ESM Fig. S2.

Although the thermodynamically stable crystalline forms of drugs are most commonly used in solid dosage forms, disordered amorphous forms of poorly soluble drugs, such as indomethacin, are of much interest to the pharmaceutical industry as a means to increase their apparent solubilities and associated dissolution rates. The time-resolved setup is capable of revealing the fine structure of both forms, whereas with the CW non-time-resolved instruments, obtaining Raman spectra for both forms was more arduous. This is of utmost importance in the pharmaceutical setting where discrimination between crystalline and amorphous forms of substances is required in order to determine phase transitions or ratios of the two forms in a mixture. Moreover, as the time-resolved setup was able to reveal even subtle Raman spectral features, differentiation between solid-state forms of compounds can be made with this novel Raman system. In the following section, we discuss in more detail the band assignments for the model drugs and describe in conceptual level some benefits that the detailed Raman spectra can offer in pharmaceutical formulation and processing contexts, as well as the novelty value of these Raman measurements compared to traditional methods and instruments.

#### Caffeine (1,3,7-trimethylpurine-2,6-dione)

Caffeine is a well-known central nervous system stimulant, found in many dietary products and occasionally used to stimulate the cardiac and bronchial systems. Caffeine is sensitive to polymorphism—and, from a pharmaceutical point of view, is an interesting compound for closer examination due to the fact that different polymorphs have potentially varying physicochemical properties as well as pharmacological activities [24]. For caffeine, both experimental and computational Raman data are well established; Fig. 2A, B; Fig. 3A; and ESM Table S1 show the measured Raman spectra and band assignments for caffeine, respectively. In the present study, caffeine was chosen to act as an easily measured reference drug. As expected, spectra acquired by CW and time-resolved setups clearly show the bands, and the peaks are in accordance between the instruments and with the DFT-based calculated values that can be found in the literature [24, 25]. The contributions from C–C, C–N, C=N, and C=O bonds can be easily used in, e.g., chemometric analysis.

#### Ranitidine HCl (*N*-[2-[[[5-[(dimethylamino)methyl]-2-furanyl]methyl]thio]ethyl]-*N*-methyl-2-nitro-l,l-ethenediamine hydrochloride)

Ranitidine HCl is a widely used gastric acid inhibitor, acting as an H2 receptor antagonist. Ranitidine HCl has two major polymorphs, form I and form II [26]. Different solid-state forms or polymorphs of the same compound exhibit different pharmaceutically important physical properties such as crystal habit (morphology), melting point, and aqueous solubility. Therefore, identification and characterization of the solid-state forms is important. In the case of ranitidine HCl, form II exhibits favorable filtration and drying characteristics over form I due to morphological differences, and this practical advantage was the basis for its much disputed patent upon its serendipitous discovery by Glaxo Group Ltd in 1981. Part of the patent disputes focused on difficulties in analysis of the solid-state forms. Raman spectroscopy has since been shown to be a convenient technique for this purpose [27]. Here, we present for the first time the quantum chemical calculations to determine Raman peaks for ranitidine HCl. On the basis of computational chemistry we performed, the furan ring and CH_2_ (1170 cm^−1^), the CH_2_ groups (asymmetric rock at 1254 cm^−1^ and wag 1301 cm^−1^), furan (symmetric stretch at 1533 cm^−1^), and the CH_2_SCH_2_ group (symmetric wag at 1276 cm^−1^) contribute to the Raman spectrum of ranitidine HCl. According to Fig. 2C, D; Fig. 3B; and ESM Table S2, the CW 785-nm Raman system has very limited capability to resolve interpretable peaks, whereas the CW 532-nm and the time-resolved CMOS SPAD instruments accomplish better to provide peaks that are relatively well in line with the DFT calculated values. However, these observations begin to show the limitations of conventional CW Raman setups; one has to rely on multiple-wavelength systems to measure various samples, which is not often feasible or even possible. Interestingly, the Raman peak at 1185 cm^−1^ has been evaluated to be characteristic of ranitidine HCl polymorphic form II whereas the peak at 1208 cm^−1^ would describe the existence of form I [28]. In Fig. 3B, 1186 and 1206 cm^−1^ exhibit shoulders of other peaks, suggesting the existence of both polymorphic forms in the sample. Furthermore, if the time-resolved representation of the 2D spectrum, in Fig. 3B (black line showing the acquired raw data), was chosen to be constructed from a slightly different time point, the feature at 1186 cm^−1^ appeared more dominant (data not shown), and the signal was not swamped by the otherwise strong fluorescent background, leading to the hypothesis that form II was dominant in the sample. This observation shows an important feature which is available with the time-resolved CMOS SPAD technique. Namely, by varying the time point (sampling point), from which the 2D Raman spectrum is constructed, it is possible to utilize the acquired raw data without further processing. A CW system at 532 nm performed better over the CW 785-nm system in the case of ranitidine HCl, but in general, the apparent fluorescence rejection factor is very low, even with the time-resolved Raman setup. It is worth stressing that the relatively low fluorescence rejection factor might be due to a short lifetime fluorescent impurity contribution that is difficult to sort out regardless of the system used. Nonetheless, compared to the conventional CW systems, which have no possibility to examine the spectra in the time dimension and at different time points after the laser shot, the potential of this time-resolved technique becomes clearer.

#### Indomethacin (crystalline) (2-[1-(4-chlorobenzoyl)-5-methoxy-2-methylindol-3-yl] acetic acid)

Indomethacin is a non-steroidal anti-inflammatory drug. The crystalline γ-form is the most stable form of indomethacin at room temperature, and it exists as dimers in this solid-state form [29]. In the present study, Fig. 2E, F; Fig. 3C; and ESM Table S3 show the experimental and calculated data for crystalline indomethacin. Variations between the measured and calculated values exist for some peaks; however, the same trend can be observed in an earlier study [30]. Nonetheless, the measured positions for the peaks with the CW Raman systems and the time-resolved setup match up relatively well, and the characteristic chlorobenzene and indole ring contributions as well as oxygen and nitrogen containing stretching vibrations are clearly observed in the spectra.

#### Indomethacin (amorphous)

For poorly water-soluble drugs, the amorphous form has a significant advantage over the crystalline form, with a higher dissolution rate and apparent solubility, but the downside is reduced stability during processing and storage [31]. Thus, e.g., the manufacturing process involving amorphous forms can be hard to control. Raman spectroscopy has proven to be the most sensitive method for the investigation of various amorphous forms of indomethacin when the forms have been studied with different techniques and multivariate analysis [32]. However, a 1064-nm FT-Raman accessory has been a prerequisite to acquire an interpretable Raman spectrum without prior bleaching for amorphous indomethacin. Figure 2G, H; Fig. 3D; and ESM Table S4 show the experimental and calculated data for amorphous indomethacin. Herein, with the 785-nm CW Raman system, a useful Raman spectrum was practically impossible to acquire. The CW experiment at 532-nm excitation gave quite visible Raman peaks; however, the sample was not chemically stable with 5-s integration times, leading to a dramatically raised baseline and subsequent saturation of the detector due to photoluminescent degradation products. Thus, the integration time of 2 s was chosen to be used with the CW 532-nm setup.

When using the 532-nm excitation laser and time-resolved detection, we are able to acquire a detailed Raman spectrum for amorphous indomethacin. In an attempt to avoid the laser-induced sample degradation, the galvo-mirror arrangement was used to move the laser beam spot on the sample constantly. Thus, we get the advantage of the short-wavelength excitation source giving rise to higher Raman scattering intensities while fluorescence is simultaneously efficiently suppressed by the time-resolved technique. A similar inconsistency between DFT calculated peak values and measured peak values is seen as in the case of the crystalline form of indomethacin. However, the spectra in Fig. 3C, D reveal detailed features that can be conveniently utilized to investigate crystalline and amorphous forms of indomethacin. For instance, the region between wavenumbers 1080–1450 cm^−1^ shows clear shifts in peak intensities, shapes, and positions. Similar differences are also seen in the range of 1500–1700 cm^−1^.

The acquisition of accurate Raman spectra of both forms can be performed with effective fluorescence suppression without the need to switch between systems or perform excessive data processing. Therefore, the time-resolved technique presented here could open up new possibilities, e.g., in analyzing the phase transition processes in pharmaceutical settings and investigating compounds that are known to be laborious to measure with Raman instruments due to weak signals and interfering fluorescence backgrounds.

#### Preliminary perspectives on the capabilities of the time-dimension analysis

The current time-gated measurement system has two main capabilities: first, rejection of sufficiently long-lived fluorescence or other background radiation from a Raman signal, and on the other hand, measuring the spectrally resolved fluorescence decay shape. We applied the previously described single-exponential tail fitting to the studied samples. Now, since caffeine does not have a fluorescence background to speak of, and ranitidine HCl has very short fluorescence lifetime, the only meaningful results are found in indomethacin. However, in the case of ranitidine HCl, even with the time-resolving capacity in the present study being restricted by the compound’s short fluorescence lifetime and thus the setup’s time resolution, we were still able to make an estimate of the fluorescence lifetime of ranitidine HCl. Namely, we could tell whether the fluorescence lifetime was short or long—a feature not available on conventional Raman setups. The fitted, uncorrected lifetimes for indomethacin are about 2.1 ns for crystalline indomethacin and 2.3 ns for amorphous indomethacin. According to a rough simulation model we performed, the actual, de-convolved lifetimes are approximately 100–200 ps shorter (data not shown). The exponential fittings on the measured data are shown in ESM Fig. S1. The capabilities of the system to measure general fluorescence decay shapes (e.g., find parameters of multi-exponential decays) will be expanded in the future. This involves determination of the instrument response function (IRF) in the sense that its effects can be de-convolved. For measuring the actual vibrational dynamics associated with the Raman processes [12, 19], neither the time nor the wavelength resolution of our system is sufficient. Nevertheless, in our current data, one can detect certain ambiguities concerning the temporal shapes of certain Raman peaks; these observations merit a further study with higher temporal resolution in order to ascertain their origin and significance.

## Conclusion

We have shown the potential of fast, picosecond pulsed 532-nm laser excitation coupled with a fast and sensitive CMOS SPAD detector to overcome the known Achilles heel, fluorescence, in Raman spectroscopy. In addition, compared to the conventional methods, we are potentially able to have more detailed structural information on our sample molecules with Raman spectroscopy by utilizing the efficiently fluorescence-suppressed and accurate Raman spectra, and the time-domain detection capability. The technical advantages of this CMOS SPAD detector have been previously discussed elsewhere [16]. Now, we demonstrate for the first time a step towards applications with this time-resolved Raman system. We record fluorescence-suppressed Raman spectra of solid, amorphous and crystalline, and non-photoluminescent and photoluminescent drugs such as caffeine, ranitidine hydrochloride, and indomethacin (amorphous and crystalline forms). The raw data acquired by utilizing only the picosecond pulsed laser and a CMOS SPAD detector proved useful for identifying the compounds directly without any data processing. Relatively straightforward algorithms can further diminish the fluorescence contribution in the acquired spectra, showing potential for accurate quantitative analysis. In summary, our major findings were as follows. Firstly, by utilizing the high photon energy 532-nm pulsed excitation source, we are able to observe accurate Raman spectra features. The quantum mechanical calculations for one of our model drugs, ranitidine hydrochloride, the comparison to existing literature, and experiments performed with CW 785- and 532-nm Raman setups verified this. Secondly, we are able to diminish the strong fluorescence backgrounds, especially on amorphous indomethacin. The findings with our model drugs indicate the applicability of this time-resolved Raman technique to overcome the ever-present problems of weak signal and fluorescence in Raman spectroscopy—not only in pharmaceutical settings but also in many other fields that would benefit from the accurate chemical information Raman spectroscopy offers. Several applications in the field of pharmaceutical industry and bio- and life sciences would benefit from the time-domain assays with Raman spectroscopy. For example, phase transition processes, cellular uptake studies, and cell culture studies regarding changes of the chemical concentrations in the culture medium or nanoparticle uptake studies would be potential areas for future research. Another potential option for further studies would be an investigation of several drug molecules in amorphous and crystalline solid-state forms. The setup described is potentially a powerful and convenient tool to overcome current fluorescence-related limitations of Raman spectroscopy for laboratory and off-laboratory use, while simultaneously providing complementary information for Raman measurements in the form of time-domain analysis. The advantages of using this CMOS SPAD technique over the existing instruments (for example [17, 18]) are practical and robust operation of the system, compact size, and affordable cost. In the case of varying samples in terms of fluorescence interference, the CMOS SPAD setup can be feasibly used for a range of samples without the need to change from one instrument to another. Thus, it offers a very potential choice of instrument for the field of Raman spectroscopy. Having showed that this new Raman technique effectively suppresses fluorescence, we envisage that it has also widespread application in different pharmaceutical and biopharmaceutical contexts that have previously suffered from fluorescence phenomena.

## Electronic supplementary material

ESM 1 (PDF 484 kb)
