# Supplementary material for: Fluorescence-suppressed time-resolved Raman spectroscopy of pharmaceuticals using complementary metal-oxide semiconductor (CMOS) single-photon avalanche diode (SPAD) detector
Source: Anal Bioanal Chem. 2015 Nov 9;408:761–74. doi: 10.1007/s00216-015-9156-6 (PMC4709379; doi:10.1007/s00216-015-9156-6)
Supplement: Supplementary file 1 — (PDF 484 kb) [file 216_2015_9156_MOESM1_ESM.pdf]

Analytical and Bioanalytical Chemistry

Electronic Supplementary Material

Fluorescence-suppressed time-resolved Raman spectroscopy of pharmaceuticals using complementary metal-oxide semiconductor (CMOS) single-photon avalanche diode (SPAD) detector

Tatu Rojalin, Lauri Kurki, Timo Laaksonen, Tapani Viitala, Juha Kostamovaara, Keith C. Gordon, Leonardo Galvis, Sebastian Wachsmann-Hogiu, Clare J. Strachan, Marjo Yliperttula

Table S1 Selected peak assignments for caffeine

CW 785 nm (cm^{-1})	CW 532 nm (cm^{-1})	Time-resolved (cm^{-1})	DFT values (cm^{-1})	Predicted description
927	928	928	927	imidazole in-plane ring deformation [1]
1071	1071	1070	1073	C-C stretching + C-N stretching [1]
1240	1239	1240	1241	C-C stretching + C-N stretching [1]
1600	1599	1595	1599	C=N, C-N stretching + CH_3 symmetric bending [1]
1656	1654	1650	1654	C = C stretching [1]
1698	1696	1691	1698	C = O stretching [1]

Table S2 Selected peak assignments for ranitidine hydrochloride

CW 785 nm (cm^{-1})	CW 532 nm (cm^{-1})	Time-resolved (cm^{-1})	DFT values (cm^{-1})	Predicted description
1186	1185	1186	1170	Furan ring and CH_2
-	-	1206	1203	CH_2 symmetric rock with furan ring
-	1259	1262	1254	CH_2 asymmetric rock
-	1274	1274	1276	CH_2SCH_2 symmetric wag
-	1301	1302	1301	CH_2 wag
-	-	1524	1533	Furan symmetric stretch

Table S3 Selected peak assignments for indomethacin (crystalline)

CW 785 nm (cm^{-1})	CW 532 nm (cm^{-1})	Time-resolved (cm^{-1})	DFT value (cm^{-1})	Predicted description
1088	1086	1085	1074	C-C stretching, in-plane chlorobenzene ring breathing [2]
1222	1219	1218	1222	C-C stretching, out-of-phase C-O-C stretching, out-of-phase C-N-C stretching (mainly dimer) [2]
1263	1264	1262	1254	Out-of-phase C-N-C stretching (monomer) and out-of-phase N-C-C stretching (dimer), out-of-phase C-O-C stretching, in-plane indole and chlorobenzene ring deformations, C-O stretching (dimer) [2]
1396	1394	1394	1408	C-C stretching, in-phase C-H inversion [2]
1588	1590	1584	1595	Chlorobenzyl ring deformation, C-C stretching [2]
1620	1618	1614	1615	C-O stretching, indole ring deformation [2]
1698	1698	1691	1705	C=O stretching [2]

Table S4 Selected peak assignments for indomethacin (amorphous)

CW 785 nm (cm^{-1})	CW 532 nm (cm^{-1})	Time-resolved (cm^{-1})	DFT value (cm^{-1})	Predicted description
-	1091	1089	1074	C-C stretching, in-plane chlorobenzene ring breathing [2]
-	1222	1219	1222	C-C stretching, out-of-phase C-O-C stretching, out-of-phase C-N-C stretching (mainly dimer) [2]
-	1259	1258	1254	Out-of-phase C-N-C stretching (monomer) and out-of-phase N-C-C stretching (dimer), out-of-phase C-O-C stretching, in-plane indole and chlorobenzene ring deformations, C-O stretching (dimer) [2]
-	1394	1390	1408	C-C stretching, in-phase C-H inversion [2]
-	1590	1584	1570	In-plane indole ring deformation [2]
-	-	1606	1615	C-O stretching, indole ring deformation [2]
-	1670	1673	1705	C=O stretching [2]

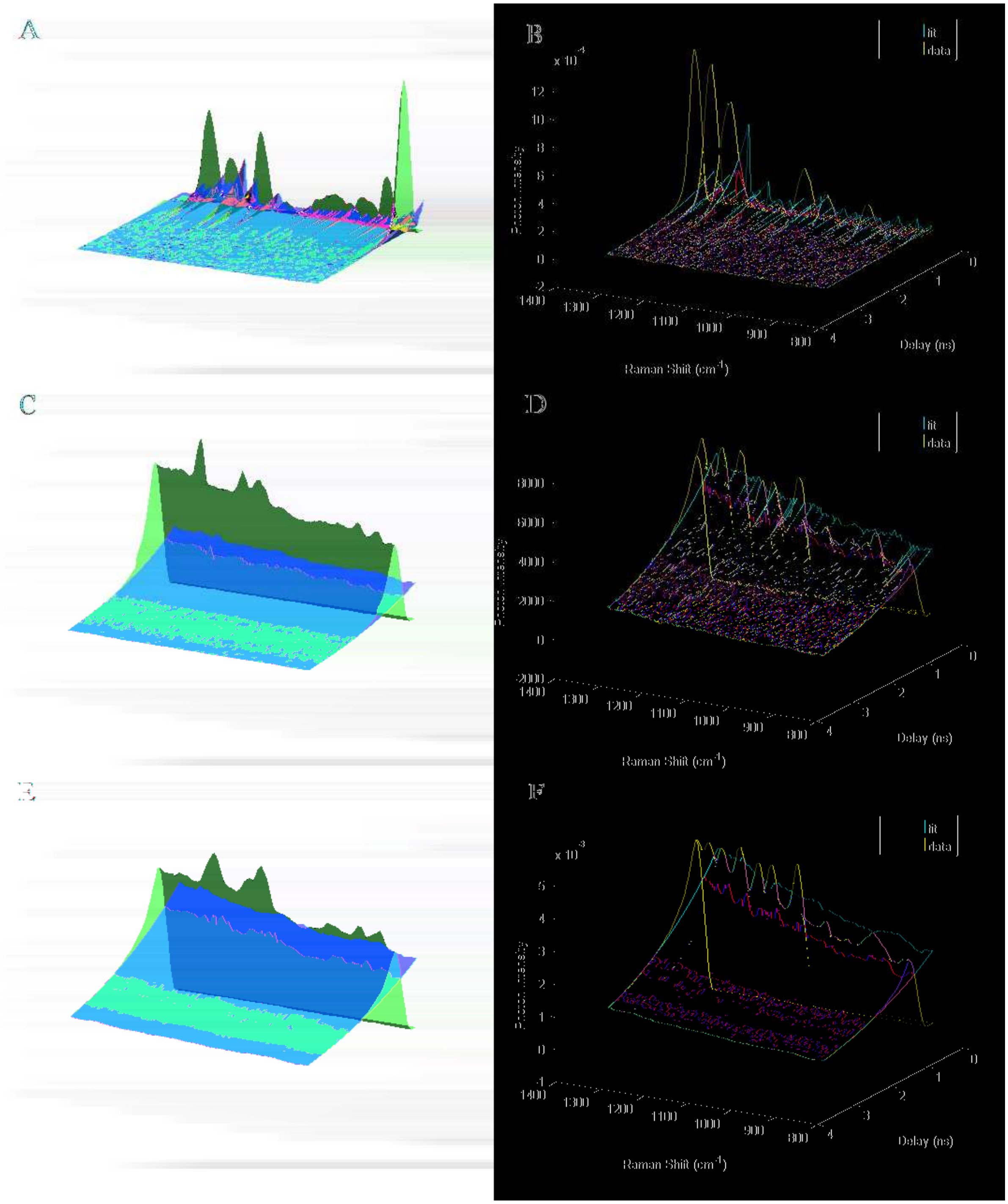

Fig. S1 Single exponential fittings on the response functions for the fluorescence decay estimations. Panels: A) caffeine (wavenumber range 1800 cm^{-1}-1300 cm^{-1}), B) caffeine (1300 cm^{-1}-800 cm^{-1}), C) crystalline indomethacin (1800 cm^{-1}-1300 cm^{-1}), D) crystalline indomethacin (1300 cm^{-1}-800 cm^{-1}), E) amorphous indomethacin (1800 cm^{-1}-1300 cm^{-1}), F) amorphous indomethacin (1300 cm^{-1}-800 cm^{-1})

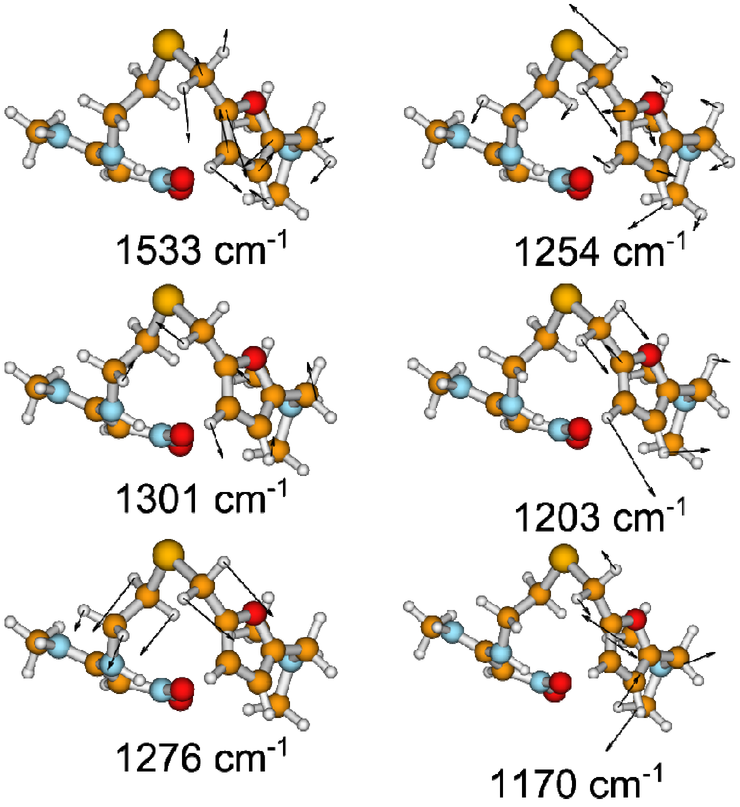

Fig. S2 Eigenvector diagrams for prominent modes of ranitidine HCl (modeled as ranitidineH^+). Values are calculated frequencies as presented in Table S2

References

1. Nolasco MM, Amado AM, Ribeiro-Claro PJ (2006) Computationally-assisted approach to the vibrational spectra of molecular crystals: study of hydrogen-bonding and pseudo-polymorphism. ChemPhysChem 7 (10):2150-2161. doi:10.1002/cphc.200600308

2. Strachan CJ, Rades T, Gordon KC (2007) A theoretical and spectroscopic study of gamma-crystalline and amorphous indometacin. J Pharm Pharmacol 59 (2):261-269. doi:10.1211/jpp.59.2.0012
